# Taking stock: understanding key processes in the public health programs—stages of research and evaluation framework

**DOI:** 10.1093/heapro/daaf213

**Published:** 2025-12-11

**Authors:** Karen Lee, Philayrath Phongsavan, Luke Wolfenden, Rachel Tabak, Adrian Bauman

**Affiliations:** Prevention Research Collaboration, Sydney School of Public Health, Faculty of Medicine and Health, The University of Sydney, Camperdown, NSW 2006, Australia; Charles Perkins Centre, The University of Sydney, Camperdown, NSW 2006, Australia; Prevention Research Collaboration, Sydney School of Public Health, Faculty of Medicine and Health, The University of Sydney, Camperdown, NSW 2006, Australia; Charles Perkins Centre, The University of Sydney, Camperdown, NSW 2006, Australia; School of Medicine and Public Health, The University of Newcastle, Callaghan, NSW 2308, Australia; School of Public Health, Washington University in St. Louis, St. Louis, MO 63130, United States; Prevention Research Collaboration, Sydney School of Public Health, Faculty of Medicine and Health, The University of Sydney, Camperdown, NSW 2006, Australia; Charles Perkins Centre, The University of Sydney, Camperdown, NSW 2006, Australia

**Keywords:** research translation, implementation science, implementation research, scale-up, scalability, sustainability, research and evaluation framework

## Abstract

The complexity of research translation is well recognized. Efforts to accelerate the process have emerged through implementation science, scale-up, scalability, and sustainability. This area of research is now inundated with a plethora of new concepts, creating overlap and confusion for those working in public health, implementation science, and scale-up as well as those seeking to understand it. This perspective examines the commonly used processes (scale-up, scalability, transferability, sustainability) and articulates their current definitions found in the literature. An explanation of the similarities and differences is also provided along with an illustration of where they may be applied in the public health programs: stages of research and evaluation framework. This perspective aims to clarify these processes, in order to provide guidance for seeking to engage in this area, how and when those processes might apply for those new to the field as well as those working within it.

Contribution to Health Promotion

- This perspective supports public health and health promotion researchers and practitioners navigate key processes of scale-up, scalability, implementation, and sustainment by identifying common confusions and clarifying them within the public health programs: stages of research and evaluation framework.
- This perspective recommends consistency in the use of these processes as their definition can influence their application in research and practice.

## PERSPECTIVE

Evidence-based practice to achieve enhanced population health outcomes is the primary goal of research translation (Rychetnik *et al*. 2012). However, research translation is often nonlinear and is limited by sub-optimal implementation, adoption, and the uptake of interventions (Lang *et al*. 2007). Furthermore, research translation has also been hypothesized to take as long as 17 years (Balas and Boren 2000). Continued efforts to expedite research translation have driven the emergence of new disciplines and processes to improve the uptake of evidence-based health interventions in the real-world context to deliver population health benefits. These include understanding implementation strategies that increase uptake and/or adoption of effective interventions in practice (Bauer and Kirchner 2020).

The emergence of “implementation science” as a new discipline has contributed to improving the uptake of evidence-based interventions (Colditz 2012, Proctor *et al*. 2013). However, this has led to increases in a variety of other related processes, such as replication and scale-up as examples being used, as evidenced through publication titles and abstracts over the last 20 years since 1995 (Klaic *et al*. 2022). Consequently, variable and inconsistent use across geographical regions and sub-specialities within health research has created confusion, making it harder for researchers to determine which of these processes are appropriate for their goals or studies (Niang *et al*. 2023). This problem is not unique to any one discipline with others also echoing our concerns that the variations in use of terminology “introduces barriers to the accessibility and usability of research findings” (Lizarondo *et al*. 2025). Such complexity and confusion can arguably detract from advancing the research translation process and implementation science field as a discipline. With this in mind, it is timely to harmonize concepts and their definitions to improve consistency and facilitate a shared understanding among researchers, policymakers, or practitioners working in this area.

Before we begin, we acknowledge the following.

Firstly, we perceive the scale-up process as a continuum, on a trajectory of the public health programs: stages of research and evaluation framework based on the model by Bauman and Nutbeam (2022). For example, any process or activity undertaken following efficacy or effectiveness testing of an intervention can be seen as being on the continuum toward “scale-up,” including if they are initially “replicated” in new locations or different target populations. Unfortunately, there is little consensus in the literature as to what magnitude of scale that is and how many sites or locations an intervention has to have been implemented in for it to be constituted as “scaled-up.” We interpret the World Health Organization’s (WHO) definition of “scale-up” to include discernible reach into whole populations either at community (local), city, state, or country levels, though this is not universally applied in the research literature (ExpandNet 2009).

Secondly, we acknowledge there have been previous attempts to facilitate a shared understanding of implementation science and scale-up. For example, articulating the similarities and differences between implementation science and scale-up (Lee *et al*. 2024); providing a plain language explanation of key concepts in implementation science using visual aids (Curran 2020) and delineating the differences between “spread,” “scale,” and “sustain” (Côté-Boileau *et al*. 2019, Wolfenden *et al*. 2024).

Thirdly, we feel it is important to distinguish between “discipline of study” and the “processes” within them. “Implementation science” is considered a “discipline of study” (Beidas *et al*. 2022), emerging in the last two decades (Bauer *et al*. 2015). It meets the criteria of a discipline as it is a bounded set of activities which includes specific theories and concepts, terminologies, research methods, and institutional manifestations (Krishnan 2009). In contrast, “scale-up,” “scalability,” and “sustainability” are generally considered as “processes” (Shediac-Rizkallah and Bone 1998, Milat *et al*. 2013, 2015, Coviello *et al*. 2024). Scale-up, e.g., emerged in the literature as early as 1995 (Uvin 1995) from the low-middle income country context and is frequently described as the diverse “processes” or “strategies” aimed at achieving the specific end-goal of reaching more people, rather than a distinct discipline of study.

Finally, we are building on what is published in the peer-reviewed literature, rather than developing or advocating for new definitions or processes. We present this information so that readers can determine which process and associated definitions are best suited for their context and purpose.

With these in mind, this perspective seeks to:

- Present a selection of definitions of processes used across the public health programs: stages of research and evaluation framework to highlight how and where they might be similar or different.
- Use the established public health programs: stages of research and evaluation framework to depict where and when these processes and disciplines can be applied.

Importantly, we recognize that many of these processes are used outside the health sector, most often in business and agriculture, and may have different meanings in those sectors (Coviello *et al*. 2024, Martinez-Baron *et al*. 2024). However, for the purposes of this perspective, we have confined their use in the health sector only.

Table 1 provides a selection of published definitions. We utilized triangulation methods, initially guided by a variety of frameworks and consulting key experts in the field along with corroboration with a previous keyword search in PubMed of increasing usage by Klaic and colleagues (2022), which led to the final selection of definitions in Table 1.

**Table 1.** Frequently used process within the public health programs: stages of research and evaluation framework.

Processes	Definition(s)	Processes that have similar definitions	When is this most relevant?^a^	Reference
Adaptability/adaptation	The degree to which an intervention can be adapted, modified, or tailored to meet the needs of various contexts and populations while retaining its essential elements	–	Can occur at any time between Steps 3–5 and beyond	Chambers *et al*. (2013)
Applicability	The likelihood that an intervention could be implemented in a new, specific setting	Replication Spread Transferability Scale-out	See replication	Wang *et al*. (2006)
De-implementation	De-implementing inappropriate or ineffective health interventions is essential for re-prioritizing effective programs, maintaining public trust, minimizing patient harm, and reducing unnecessary resource waste in health care and public health	A scoping review identified 43 different terms for de-adoption (e.g. de-prescribe, abandon, de-implement)	Post-implementation/post scale-up or even post sustainment	Niven *et al*. (2015), Norton *et al*. (2017)
De-adoption	De-adoption was defined as the discontinuation of a clinical practice after it was previously adopted	De-implementation	See de-implementation	Rogers (2002)
Dissemination	The purposive and intentional process of achieving the maximum uptake of effective and feasible health interventions into a community, a stage where an intervention is scaled up to reach a large population or population group	Scale-up	See scale-up	Bauman and Nutbeam (2022)
Diffusion	Passive spread of an innovation, which is typically informal and largely uncontrolled	–	Generally associated with scale-up with some differences	Bradley *et al*. (2011), Greenhalgh *et al*. (2004)
Implementation research	Scientific methods to answer questions concerning implementation—research to identify the best implementation methods to be used in research translation	–	Can be conducted at any point across the Research translation process, generally from Steps 2–5	Peters *et al*. (2013)
Implementation science	The scientific study of methods to promote the optimal uptake of evidence-based findings into routine practice and, hence, to improve the quality and effectiveness of health services. Hence, the goal of implementation science is not to establish the health “impact” of a clinical innovation, but to identify factors that maximize its uptake.	–	Occurs after Step 3, results can be used to inform scale-up, sustainment and ongoing institutionalization	Eccles and Mittman (2006)
Institutionalization	Institutionalization: final stage, program innovations that successfully become integrated into the funding, priorities, and delivery systems of their host organizations	Sustainment Routinization	Occurs after Step 5	Steckler and Goodman (1989)
	Where a program has been successfully diffused into a community, has established policy support and funding mechanisms, and continuing community support			Bauman and Nutbeam (2022)
Mis-implementation	Refers to both the de-adoption of effective programs, policies, or other interventions that should continue and to the continuation of ineffective interventions that should end.	De-implementation De-adoption	Post-implementation/post scale-up or even post sustainment	Brownson *et al*. (2015)
Replication	Process of evaluating an intervention when conducted in a different setting or with a different population or subgroup to assess whether intervention effects are similar or different to the original efficacy study	Scale-out Transferability Spread	Occurs between Steps 3 and 4, that is, after evidence generation and usually before scale-up	Bauman and Nutbeam (2022)
Research translation	The process where empirical approaches build on new types of evidence, and where the process of translation is tested through replication and dissemination of interventions previously shown to be effective.	–	Includes Steps 1–5	Rychetnik *et al*. (2012)
Routinization	How innovations become part of standard practice	Institutionalization Sustainment	See institutionalization	Yin (1981)
Scalability	The ability of a health intervention shown to be efficacious on a small scale and or under controlled conditions to be expanded under real-world conditions to reach a greater proportion of the eligible population, while retaining effectiveness.	Transferability Replication Applicability	Should be planned from Step 2, but generally occurs between Step 3 and 4. Describes a process that occurs between Steps 3 and 5	Milat *et al*. (2013)
	The potential for “extending the reach of an intervention by replicating it in other localities, cities or states (horizontal scale up)”			Reis *et al*. (2016)
Scale-up Scaling-up	“Deliberate” efforts to increase the impact of “successfully tested” health interventions to “benefit more people” and to foster policy and program development on a “lasting basis”	Spread Dissemination	Step 4—occurs after evidence generation (Step 3)	WHO (2010)
	Deliberate effort to broaden the delivery of an evidence-based intervention (EBI) with the intention of reaching larger numbers of a target audience within the same, or very similar settings, under which the EBI has already been tested			Aarons *et al*. (2017)
	Tackling the problems across a system that arise during full-scale implementation			Greenhalgh and Papoutsi (2019)
Scale-out	…the deliberate use of strategies to implement, test, improve, and sustain an EBI as it is delivered to new populations and/or through new delivery systems that differ from those in effectiveness trials.	Replication Transferability Spread Applicability	Occurs after Step 3 but prior to Step 4	Aarons *et al*. (2017)
Spread	Replicating an initiative somewhere else	Replication	–	Greenhalgh and Papoutsi (2019)
	Passive and/or deliberate efforts to communicate and implement an innovation and usually involves adapting an innovation to a new setting		–	Côté-Boileau *et al*. (2019)
Sustainability	Defined as (i) after a defined period of time, (ii) a program, clinical intervention, and/or implementation strategies continue to be delivered, and/or (iii) individual behavior change (i.e. clinician, patient) is maintained; (iv) the program and individual behavior change may evolve or adapt while (v) continuing to produce benefits for individuals/systems.	Sustainment	Generally is achieved after Step 4, scale-up. However can also occur after Step 3 (evidence generation). However, should be considered from Step 2	Moore *et al*. (2017)
	Continued use of intervention components and activities for the continued achievement of desirable health outcomes within the population of interest			Scheirer and Dearing (2011)
Sustainment	Refers to the sustained use or delivery of an intervention following [withdrawal of] external implementation support	Sustainability	See sustainability	Shelton *et al*. (2018), Aarons *et al*. (2011)
	Refers to the continued enactment of processes, practices, or work routines that are conveyed and learned through an intervention			Fleiszer *et al*. (2015), Chambers *et al*. (2013)
Transferability	The likelihood that the study’s findings could be replicated in a new, specific setting (i.e., that its effectiveness would remain the same)32	Scalability Replication Spread Applicability Scale-out	See replication	Wang *et al*. (2006)

^a^For steps, see Fig. 1 (numbered circles).

**Figure 1.**
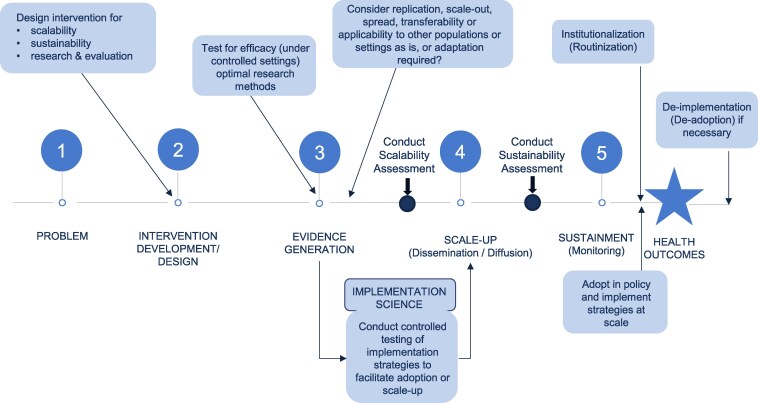
Key steps and activities within the public health programs: stages of research and evaluation framework (adapted from Bauman and Nutbeam 2022).

Figure 1 illustrates where these processes may be relevant in the overall public health program evaluation planning cycle.

### Scale-up, dissemination, and diffusion

There are a variety of published definitions of “scale-up” that mostly describe it as a “process” (of reaching more people or organizations with an intervention). There is a general consensus that scale-up comprises “deliberate,” “conscious,” and purposive actions, which differ from the passive process of program “diffusion,” which happens organically (ExpandNet 2009).

Scale-up is considered a continuous “process,” whereby steps are deliberately and gradually taken to expand the intervention to more people (WHO 2010). The WHO delineates a variety of approaches to scale-up, such as “spontaneous scale-up” which is much like “diffusion,” along with vertical and horizontal scale-up (WHO 2010). Vertical scale-up occurs when an intervention is simultaneously introduced across the whole system (top down), while horizontal scale-up tends to refer to a more incremental and/or staged approach to scale-up (WHO 2010). There are some who consider that “scale-up” is an “outcome” where the “outcome” for an intervention is to be “scaled-up” (Bulthuis *et al*. 2020). However, whether “scale-up” is an outcome or process and is likely dependent on the intervention and/or context (Côté-Boileau *et al*. 2019), as scale-up occurs on a continuum and there is no universal agreement on the minimum number of organizations or locations an intervention has to be implemented in for it to be considered “scaled-up.”

“Scale-up” is often used interchangeably with “dissemination,” an analogous process. The choice of which term to use appears to be geographically and culturally specific. In the USA, the National Institutes of Health uses “Dissemination and Implementation (D&I) Research” to describe much of its scale-up efforts (Adult & Child Center for Outcomes Research & Delivery Science (ACCORDS): Dissemination and Implementation Science Program 2021, National Cancer Institute 2022), while in Australia, Canada, and many other countries, the term “scale-up” is more prevalent (Milat *et al*. 2014, Ben Charif *et al*. 2017).

Importantly, some suggest that scale-up goes beyond “replication” (another process discussed later) as scale-up is multidimensional and multidisciplinary occurring across complex political, cultural, economic, and institutional contexts (Simmons and Shiffman 2007) while replication tends to be more limited in its application.

Finally, “scale-up” and “sustainability” are often studied together but have different steps, frameworks, indicators, and methods, and both could be considered from the planning stages of development but are conducted at different points in the program evaluation and planning cycle (Simmons and Shiffman 2007, Shelton *et al*. 2018) (see Fig. 1).

### Scalability

Scalability is the “potential” for an efficacious intervention to be scaled up (Milat *et al*. 2013). This definition of scalability by Milat *et al*. (2013) implies that “assessments of scalability” are “point in time” deliberations that should be undertaken when considering if an intervention can be scaled-up; this is best conducted after the intervention effectiveness has been demonstrated (Milat *et al*. 2020, Ben Charif *et al*. 2022).

However, there are variations to the Milat et definition, e.g., Reis *et al*. (2016) defined scalability as the potential for “extending the reach of an intervention by replicating it in other localities, cities or states (horizontal scale up)” (Reis *et al*. 2016). This Reis *et al*. (2016) definition may be confusing, as it conflates other processes on the continuum, namely, “horizontal scale-up” and “replication.” “Scalability” is sometimes conflated with “transferability” and/or “applicability” (Ben Charif *et al*. 2022); however, it has some important differences which are discussed further below.

### Sustainability, sustainment, and institutionalization

The definitions of “sustainability” and/or “sustainment” are also subject to much debate along with a lack of consensus, whether they even warrant delineation. With respect to the former, Stirman *et al*. (2012) suggested that an intervention could be considered sustained at a given point in time if, after the initial implementation support had been withdrawn, core elements were maintained along with adequate capacity for their continued implementation. Others have indicated that sustainability needs to include additional elements such as outcomes also being maintained (Moore *et al*. 2017). Sustainment, frequently defined as the “continued delivery of processes, practices or work routines delivered through the intervention” (Fleiszer *et al*. 2015) or “sustained use or delivery of an intervention following (withdrawal of) external implementation support” (Shelton *et al*. 2018 ) focuses more on the delivery of the intervention rather than maintenance of health behaviors or outcomes (Wolfenden *et al*. 2024).

Additionally, much like scale-up, sustainment is sometimes considered an “outcome,” in particular, the “final outcome” of the research translation model (Dearing 2009, Scheirer and Dearing 2011) or the outcome of the process of scale-up (Proctor *et al*. 2011). Some even consider sustainment as a sub-concept within implementation, recognizing the process as being dynamic, requiring adaptation in different contexts (Shelton *et al*. 2018). Several types of sustainment strategies have been hypothesized to be prevalent, which include self-sustainment, dynamic, and static supported sustainment (Wolfenden *et al*. 2024).

Irrespective of the ongoing debate on the definitions for scalability and/or sustainability, it is important that planning for scalability and sustainability occurs concomitant with intervention and implementation strategy development (Pluye *et al*. 2004, Milat *et al*. 2020). Early planning for scalability and sustainability will enhance understanding of the factors and conditions needed to facilitate the successful scale-up and sustainment of health interventions.

Finally, the concept of “institutionalization” may have some overlap with sustainability, with one difference. “Institutionalization” means that the intervention has been integrated into routine operations and delivery systems has established policy support and funding and continuing community support (Bauman and Nutbeam 2022). However, it is possible for an intervention to be “sustained” without having established policy support or funding as implied by “institutionalization,” particularly if it is operating on a small scale only within a small number of settings/facilities. Therefore, we acknowledge that the differences in definitions imply that it is possible to sustain an intervention without ever institutionalizing it.

### Replication, transferability, applicability, scale-out, and spread

As noted in Table 1, the definitions for replication, transferability, applicability, spread, and even scale-out overlap. At their core, they all refer to delivering an evidence-based intervention in populations, settings, or delivery systems that are different from the setting where the initial effectiveness study was conducted. As an example, “spread” has been defined as “replicating the intervention elsewhere” (MacInnes *et al*. 2023). Transferability and applicability, on the other hand, specifically refer to the likelihood that the same intervention could be replicated (delivered) in a new or different setting (Wang *et al*. 2006) while scale-out adds the dimension of testing new strategies to either implement, improve, or sustain the intervention in their new population, setting, or delivery system (Aarons *et al*. 2017). The assumption here is that an intervention that works well in one area or within a given set of conditions cannot be assumed to be replicated, spread, transferred, applied, or even scaled out to a different area, population, or delivery system with the same level of success (Bauman and Nutbeam 2022).

Importantly, one difference between replication, transferability, applicability, spread, scale-out, and “scalability” (discussed earlier) is that scalability explicitly states that the aim is to “expand and increase population reach,” which implies “scale” (Milat *et al*. 2013). In contrast, transferability, applicability, replication, spread, or scale-out do not appear to have that same emphasis on achieving “scale,” though their processes are on the continuum to scale-up. This is an important distinction as to scale-up an intervention to multiple locations/settings/sites requires different considerations to replicating or transferring the intervention to a small number of new settings.

### Implementation research and implementation science

Implementation research is a discipline which encompasses activities pertaining to all aspects of the research translation process (Peters *et al*. 2013). At almost every stage of the program evaluation planning cycle, implementation can occur and can be studied, either through research, evaluation, or implementation trials. As an example, realist evaluations use mostly qualitative methods to explore how the implementation of an intervention works, in what circumstances and for whom, and considers the context, mechanisms, and implementation factors as part of its investigation (Chelimsky and Shadish 1997, Pawson and Tilley 1997). The last two decades have seen the growth of a new discipline, “implementation science,” which aims to “improve the adoption and/or uptake of an already demonstrated effective intervention through testing and optimizing implementation strategies” (Eccles and Mittman 2006). Implementation science generates evidence on the effectiveness of different implementation strategies and may be research conducted at the individual level (clinicians, practitioners, nurses) or organizational level (hospitals, community health services) (Curran *et al*. 2012). Implementation trials are usually controlled by researchers, and outcomes focus on implementation indicators, not always on health outcomes (Curran *et al*. 2012, Lee *et al*. 2024). As a discipline of study, Implementation science sits outside the continuum to scale-up but generates information that can identify optimal strategies for use in scale-up processes. Scale-up requires some type of “implementation,” but implementation trials can occur without (and prior to) scale-up (Lee *et al*. 2024). Implementation strategies can sometimes be tested in small pilot studies, without explicit intentions to scale (Lee *et al*. 2024).

### Research and evaluation

The development of an evidence base is an important part of the research translation process (Bauman and Nutbeam 2022). There is a range of research designs used in translating evidence to policy and practice, and scale-up exists within the evaluation continuum. Evaluations may also be conducted for other purposes, including accountability, program learning, or decision making as well as evidence of effectiveness (Canadian Evaluation Society 2025), and can focus on assessing the processes and/or outcomes of a program (Bauman and Nutbeam 2022). There are different types of evaluation paradigms that all bring different perspectives. For example, realist evaluation is based on the assumption that the same intervention will not work everywhere and for everyone, and evaluations need to understand what works for whom, under what circumstances, and how (Chelimsky and Shadish 1997, Pawson and Tilley 1997). Empowerment evaluations prioritize fostering engagement of key community stakeholders involved in interventions (Fetterman and Wandersman 2004), and utilization-focused evaluations focus on ensuring that evaluation findings are useful to generate relevant information (Patton 2002, Patton 2018). Scale-up actions often fit within the public health programs: stages of research and evaluation framework (Fig. 1), whereas implementation science often fits within a funded research framework.

### De-implementation, de-adoption, and mis-implementation

The processes of de-implementation, de-adoption, and/or mis-implementation are emerging in the research literature. It was recognized that some interventions are ineffective or result in negative and unintended consequences (Brownson *et al*. 2015, Norton *et al*. 2017). Brownson *et al*. (2015) described “mis-implementation” in public health practice as “both the de-adoption of effective programs, policies, or other interventions that should continue and to the continuation of ineffective interventions that should end,” which highlights a conflict often occurring in practice. Some years later, Norton and colleagues re-emphasized a similar process; however, they termed it as “de-implementation” which is the deliberate action of ceasing the operations of interventions, through their removal, replacement, reduction, or restrictions to their delivery that are ineffective (Norton *et al*. 2017, Norton and Chambers 2020). These processes all recognize that some interventions need to be actively discontinued as they are no longer effective, and the act of de-implementing ineffective interventions can be as important as implementing effective interventions.

## CONCLUSION

There is a range of processes in program evaluation with the aim of understanding research translation and scale-up of interventions in the real world to improve population health. This has led to new frameworks, models, resources, checklists, and tools to facilitate this research translation. Over time, different definitions have been conflated and caused confusion amongst those researchers, policymakers, and practitioners who seek to operationalize them in practice. This perspective describes stages of research and an evaluation framework for public health programs by clarifying their definitions. We believe that while many of the processes may overlap, their aims can be quite different. Where possible, we have illustrated their similarities and differences. Not only do we believe this perspective will help operationalizing them in practice, but it may also foster collaborations between researchers, policymakers, and practitioners to make more informed decisions on the appropriate processes to use that would best suit their context and study. Future exploration of the epistemic and ethical foundations underpinning these processes would be a valuable area for further investigation.

## Data Availability

All data are incorporated into the article.
